# Raman Mapping of Biological Systems Interacting with a Disordered Nanostructured Surface: A Simple and Powerful Approach to the Label-Free Analysis of Single DNA Bases

**DOI:** 10.3390/mi12030264

**Published:** 2021-03-04

**Authors:** Valentina Mussi, Mario Ledda, Annalisa Convertino, Antonella Lisi

**Affiliations:** 1 Institute for Microelectronics and Microsystems, National Research Council, IMM-CNR, 00133 Rome, Italy; annalisa.convertino@cnr.it; 2 Institute of Translational Pharmacology, National Research Council, IFT-CNR, 00133 Rome, Italy; mario.ledda@ift.cnr.it (M.L.); antonella.lisi@ift.cnr.it (A.L.)

**Keywords:** single DNA bases, raman mapping, silicon nanowires, principal component analysis

## Abstract

This article demonstrates the possibility to use a novel powerful approach based on Raman mapping of analyte solutions drop casted on a disordered array of Ag covered silicon nanowires (Ag/SiNWs), to identify the characteristic spectral signal of the four DNA bases, adenine (A), thymine (T), cytosine (C), and guanine (G), at concentration as low as 10 ng/µL, and to study their specific way of interacting with the nanostructured substrate. The results show a distinctive and amplified interaction of guanine, the base that is most susceptible to oxidation, with the nanostructured surface. Our findings explain the recently revealed diverse behaviour of cancer and normal DNA deposited on the same Ag/SiNWs, which is ascribed to mechanical deformation and base lesions present on the oxidised DNA molecule backbone and causes detectable variation in the Raman signal, usable for diagnostic purposes. The notable bio-analytical capability of the presented platform, and its sensitivity to the molecule mechanical conformation at the single-base level, thus provides a new reliable, rapid, label-free DNA diagnostic methodology alternative to more sophisticated and expensive sequencing ones.

## 1. Introduction

Nanomedicine—the application of nanomaterials and nanotechnologies in the diagnostic and therapeutic field—is one of the greatest scientific innovations of recent years [1,2]. Thanks to the reduced size, the high surface/volume ratio and the possibility to modify shape and surface chemistry, nanomaterials can interface and interact with biological systems and macromolecules (cells, DNA, proteins) at different scales of spatial resolution, from micro down to nanometre range, capturing physicochemical information and exchanging signals and organic matter with these biosystems. This will allow, in the very near future, the arrival of an early disease diagnosis and personalised therapies.

To date, one of the most relevant diagnostic targets is the free circulating DNA (cfDNA), which is present in the peripheral blood [3], and is also characterised by a component released by necrotic or apoptotic tumour cells (circulating tumour DNA, ctDNA). The analysis of these DNA fragments in a peripheral blood sample, also known as liquid biopsy, can influence clinical decisions, enabling the discovery of a tumour when it is not yet visible with traditional screening tools, which is a fundamental challenge in oncology [4,5]. Standard methods routinely used for DNA detection are PCR (Polymerase Chain Reaction, and its modifications) and NGS (Next-Generation Sequencing) [6,7,8]. Anyway, these sophisticated methods present various critical issues related to laborious DNA amplification procedures, which in turn lead to an increase in costs and a lengthening of analysis times and a reduced length of readable DNA fragments. Therefore, new solutions are being developed to improve or even replace PCR and NGS techniques.

Alternative techniques for label-free DNA analysis have been proposed and demonstrated based on arrays of silicon nanowires (SiNWs) as electrical transduction agents [9,10]. Such electronic-based detectors exploit conductance change upon DNA hybridisation with specific probes immobilised on the NWs surface. They, thus, require a dedicated substrate pre-functionalisation, as well as amplification of the DNA target, and complex and expensive fabrication procedures of the FET biosensing device, including several technical challenging steps related to positioning, circuiting and integration of individual nanodevices [11] or the demanding realisation of ordered arrays of nanostructures [12]. In some cases, the luminescence quenching of the NWs caused by the DNA hybridisation is analysed, with similar drawbacks [13].

Surface-enhanced Raman spectroscopy (SERS) approaches, based on metallic colloidal solutions or nanostructured surfaces, offer a powerful solution for sensing of DNA, because they enable rapid and direct detection and monitoring of the spectral characteristics of target analytes, with reduced instrumental complexity and cost, as well as sample preparation time. Among them, dense silicon nanowires (SiNWs) array coated by metal nanoparticles or thin films have emerged as promising tools for label-free analysis [14,15,16,17,18,19,20], and suitable interface for several biological targets, including mammalian cells [21,22] and biomolecules [18,20,23].

In particular, a substrate made of a disordered array of Ag-covered SiNWs (Ag/SiNWs) has been recently proved to be a very effective and robust label-free SERS platform [14]. Its sensing ability is based on both the reduced dimensionality of the NWs and their disordered arrangement—allowing, at the same time, the physical trapping of the molecules, and the generation of hotspots (light localisation) in narrow gap regions between neighbouring and crossing NWs, which produces a strong local field enhancement [18]. Thus, the Ag/SiNWs platform does not need a stringent control of the size distribution of the nanometric probe, precise tuning of the laser excitation wavelength, or an exact reproducibility of the enhancement mechanism, which are the main problems compromising a stable signal increase in most of the SERS active nanomaterials. Besides, compared to other SERS methods, the diagnostic potentiality of this system is even much broader, since it can also capture chemical and physical molecular changes at the micro-nano scales. Indeed, it is well known that the specific interaction of biomolecules with a metal nanostructured surface has a significant influence on their observed Raman response [24], and can lead to the appearance of new spectral features and to relevant changes in vibrational peak intensities associated with substrate-molecule bind formation and relative orientation. Recently, we and others have demonstrated the remarkable ability of disordered Ag/SiNWs to detect human genomic DNA and discriminate samples coming from normal and cancer cells, without analysing its complex composition or sequencing [20]. The discrimination is due to a diverse conformation and accommodation of the DNA molecules on the NWs upon dehydration of the aqueous sample droplets directly deposited onto the array, so that the diagnostic information is obtained in an easy and fast way by simply applying principal component analysis (PCA) to the spectra composing the Raman maps of the drops. As hypothesised in Reference [20], the DNA lesions present oxidation of guanine that can strongly modify the DNA backbone torsional angle. Thus, the environment around an oxoguanine-cytosine base pair will be fundamentally different from that of the native guanine-cytosine. This effect, which is prominent in cancer or oxidised DNA with more amounts of lesions, will cause a different interaction of the normal and cancer DNA with the Ag/SiNWs substrate, and a consequent relevant variation in the Raman signal usable to distinguish DNA from normal and cancer cells.

Here, we further investigate this aspect, showing the ability of this SERS platform to identify the characteristic Raman signal of the DNA building blocks, i.e., the four DNA bases, namely, adenine (A), thymine (T), cytosine (C) and guanine (G), and to analyse their specific way of interacting with the nanostructured substrate. The target solutions have been separately dropped on the Ag/SiNWs and left to dry. The spectral data have been collected by a Raman mapping procedure and analysed using PCA to reveal and study faint differences between samples. We believe that a correct assessment of these differences will permit a better understanding of the origin of different structural and conformational properties of normal and cancer DNA. Combined with the key technological benefit offered by the disordered arrangement of NWs, which is obtained through high yield and large-area bottom-up fabrication technologies at relatively low growth temperatures (350 °C), compatible with polymeric film and glassy supports [14,23,24], the proposed platform will lay the foundations for a new reliable, rapid, label-free DNA analysis methodology alternative to the more sophisticated and expensive sequencing methodology.

## 2. Materials and Methods

### 2.1. Ag/SiNWs Fabrication

Au catalysed SiNWs were produced by plasma-enhanced chemical vapour deposition (PECVD) on microscope glass slides and Si wafers. To induce the NWs growth, a 2 nm thick Au film was evaporated on the substrate. The SiNWs growth was performed with SiH_4_ and H_2_ as precursors, at a total pressure of 1 Torr and flow ratio SiH_4_/(H_2_ + SiH_4_) fixed to 1:10. The substrate temperature was kept at 350 °C. A 13.6 MHz radiofrequency with power fixed at 5 W to ignite the plasma. The Ag-coverage was obtained by evaporating an Ag film onto the SiNWs array with a nominal thickness of 100 nm.

### 2.2. Morphological Characterisation of Ag/SiNWs

The morphology of the NWs after DNA dehydration was verified by scanning electron microscopy (SEM). A ZEISS EVO MA10 SEM was used at an accelerating voltage of 5 kV. An Au layer 10 nm thick was evaporated onto the sample with DNA before the SEM observations.

### 2.3. Single-Base Sample Preparation

The dATP, Deoxyadenosine Triphosphate (18252-015 purity >99% confirmed by HPLC), dCTP, Deoxycytidine Triphosphate (18253-013 purity >99% confirmed by HPLC), dGTP, Deoxyguanosine Triphosphate (18254-011 purity >99% confirmed by HPLC) and dTTP, Deoxythymidine Triphosphate (18255-018 purity >99% confirmed by HPLC) were obtained from Invitrogen by ThermoFisher Scientific and used without further purification. Each single dNTP nucleotides were provided as a ready-to-use solution at a stock concentration of 10 mM in Tris-HCl (pH 7.5) and stored at −20 °C. For the single DNA base experiment, 100 ng of each single nucleotide was diluted in a volume of 10 µL Milli-Q water (final concentration 10 ng/µL) and dropped on Ag/SiNWs substrates. For the mix DNA base experiment 25 ng of each single nucleotide was mixed and diluted in a volume of 10 µL (final concentration 10 ng/µL) and dropped on Ag/SiNWs substrates. These experiments were performed at least 3 times with high reproducibility.

### 2.4. Raman Mapping

Raman maps have been acquired over a squared area in the central part of the drops with a Thermo Fisher DXR2xi Raman Imaging Microscope. Each map (step-size 3 µm) was composed of about 30,000 spectra, obtained with 1 mW laser power, 0.005 s of exposition and 10 accumulations. The excitation wavelength was 532 nm, and the data have been collected with a 50× objective.

### 2.5. Principal Component Analysis

PCA has been performed comparing the spectra composing the four maps on adenine, cytosine, guanine and thymine. The first three principal components, PC1, PC2 and PC3, have been computed, and the corresponding spectral loadings in the range between 50 and 1600 cm^−1^, have been evaluated.

## 3. Results and Discussion

**Figure 1:**
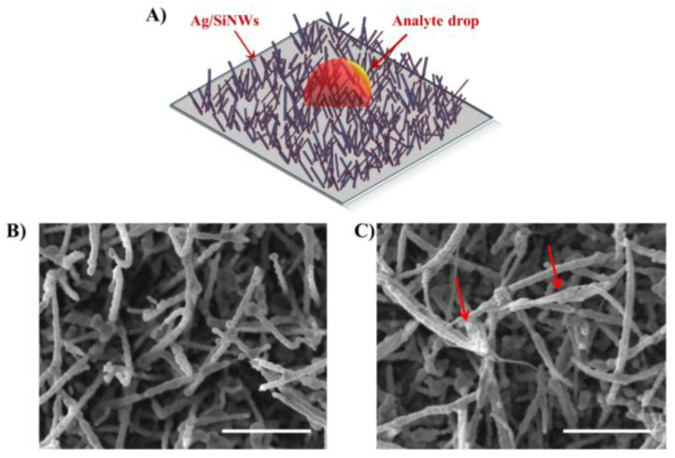
A schematic representing the analyte drop onto the array of Ag-covered silicon nanowires (Ag/SiNWs) (**A**). SEM images of the Ag/SiNWs outside (**B**) and inside (**C**) the dried droplet of the aqueous solution containing adenine. Scale bar 1 μm.

For each of the four single DNA base samples, a 10 μL drop of the aqueous solution containing 100 ng of nucleotides (concentration of 10 ng/µL) was deposited onto the Ag/SiNW substrates and left to dry for about 30 min under controlled temperature and humidity laboratory conditions, as represented by the schematic in Figure 1A. Twenty-five nanograms of each four single nucleotides were prepared and similarly deposited (concentration of 10 ng/µL).

Figure 1B,C present, respectively, SEM images of the Ag/SiNWs outside and inside the dried droplet containing adenine. They show a dense ensemble of disordered and randomly oriented NWs, 2–3 μm long and with an average radial size around 70–80 nm. SEM analysis reveals that the morphology of the Ag/SiNWs remains almost identical after sample deposition, except for the visible sticking of the NWs in some location internal to the drop (see the red arrows in Figure 1C). As elsewhere discussed [18,20], the capillary forces and surface tension during the drying process of the analyte solution cause the leaning of the nanowires, which trap the molecules and provide an efficient hotspot formation. A very similar behaviour has been observed for all the analysed samples.

**Figure 2:**
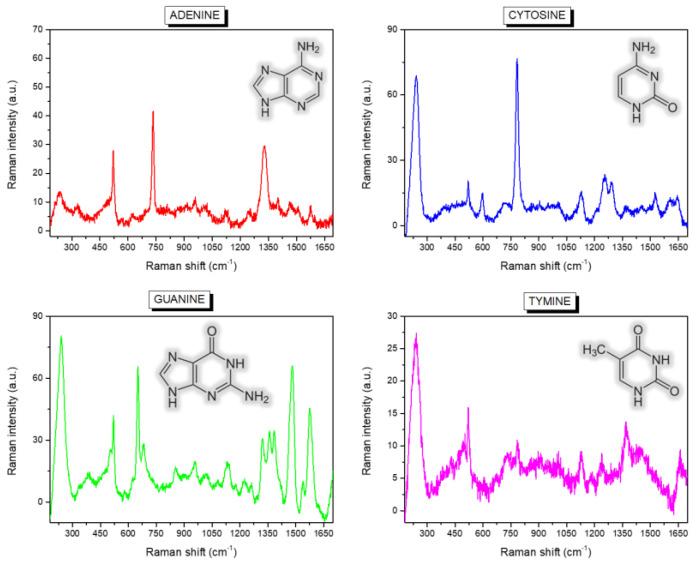
Mean Raman spectra calculated over the entire maps collected on the drops of the four single-base samples (adenine (A), thymine (T), cytosine (C) and guanine (G)). The chemical structure of the molecules is sketched as an inset of the graphs.

The region corresponding to the dried drops was then imaged by the Raman microscope collecting about 30,000 spectra for each map. Figure 2 presents the mean Raman spectra calculated over the entire maps of adenine (A), cytosine (C), guanine (G), thymine (T).

Many characteristic spectral fingerprints of the single-bases can be easily distinguished and recognised, such as the ring modes located at about 733 and 651 cm^−1^ for, respectively, adenine and guanine, and the ring breathing of cytosine at 783 cm^−1^, while the ring breathing of thymine at about 776 cm^−1^ is slightly less intense, the overall spectrum appearing less structured. The vibrational peak of silicon at about 520 cm^−1^ is also present in all the spectra, due to the contribution of the substrate itself. Table 1 summarises the identified peaks with possible assignments from refs [25,26,27].

**Figure 3:**
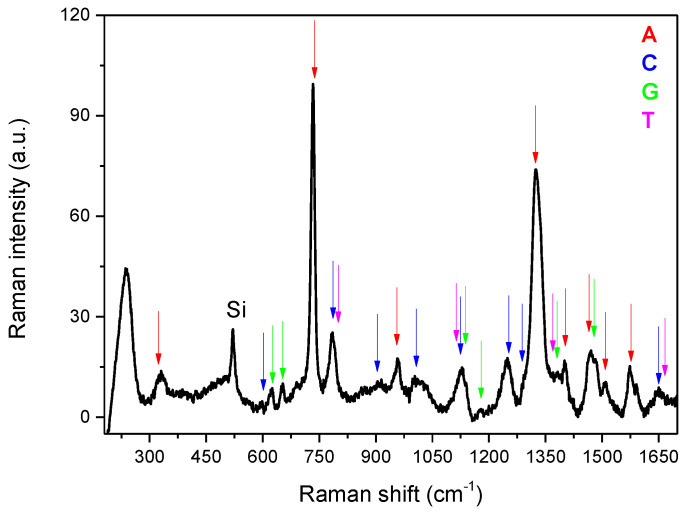
Mean Raman spectra calculated over the map collected on the mixed sample. The various bands, indicated by the arrows, are attributed to the four bases using a colour code.

Moreover, the spectrum corresponding to the mixed sample presented in Figure 3 clearly shows a correct superposition of all the various bands, indicated by the arrows and attributed to the four bases using a colour code, making it possible to conceive the method to also analyse specific DNA sequences.

On the contrary, no Raman signal was detected when the solutions are dropped on a control planar Ag/Si substrate, which proves the unique capability of the nanostructured platform to largely enhance and reveal the signature of the target nucleotides for concentrations as low as 10 ng/µL. In this regard, the disordered arrangement of the metal covered nanostructures gives a further advantage: Usually, the intensity of the Raman bands in SERS spectra of nucleotides critically depends on the positioning and geometry of the molecule with respect to the silver surface [27]—which is not a limit in our case due, to the possibility of mapping a large area containing thousands of differently oriented nanowires in direct contact with the deposited and dried nucleotides. It is also worth noting that this very simple drop-casting method is competitive with respect to other highly sensitive SERS based investigations devoted to single DNA base analysis [28].

**Figure 4:**
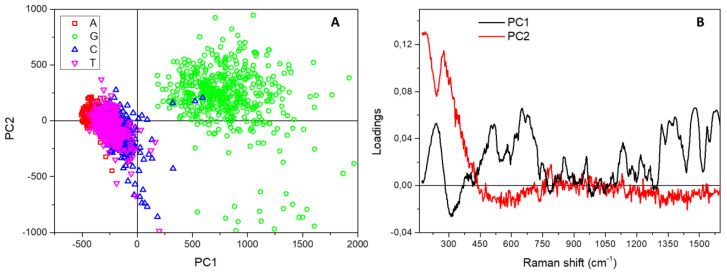
(**A**) Score plot resulting from the Principal Component Analysis performed on the spectra composing the Raman map acquired on adenine (red squares), cytosine (blue circles), guanine (green upward triangles) and thymine (magenta downward triangles) samples. (**B**) Spectral loadings corresponding to the first two principal components.

A further relevant band, at about 230 cm^−1^, is visible in Figure 2, which is not characteristic of solid or solution spectra of DNA bases, but is ascribable to the formation of an Ag-N bond between the biological molecules and the metal surface [27,29,30,31,32]. Even if its intensity is quite variable for the four bases, the peak is present in all the studied samples and the mixed one. As we have recently demonstrated [20], this important spectral feature also accounts for the different arrangement and structural conformation of healthy and malignant DNA on the SiNWs platform, and can be, thus, successfully used for direct diagnostic purposes. In this context, following the interpretation reported in Reference [33], we suppose that a fundamental role is played by the different chemical environment around nucleobase lesions or oxidation in cancer cell DNA, which in turn causes a variation in the nearby electronic cloud and backbone deformation during the conformational modifications driven by the dehydration process. To evidence a possible diverse behaviour of the four nucleotides during the drop drying, we used PCA to collectively analyse and compare the spectra composing the four Raman maps. Figure 4A shows the resulting PC scores for the first two principal components corresponding to the many point spectra coming from the map of adenine (A, red squares), cytosine (C, blue circles), guanine (G, green upward triangles) and thymine (T, magenta downward triangles) drops.

While the clouds associated with A, C and T samples appear substantially superposed, the cluster formed by data points obtained on G are separated and displaced on the PC1 and PC2 positive half-planes. In fact, the loadings of the two first principal components reported in Figure 4B show that the displacement of the G cloud along PC1 directly relates to the characteristic and intense spectral peaks appearing in the mean Raman spectrum of guanine (see Figure 2), while the shift up along PC2 is directly and specifically correlated with the band at 230 cm^−1^ due, as said, to the formation of a direct bond between the Ag-covered substrate and the nucleotides. The data, thus, reveal a distinctive and amplified interaction of guanine with the nanostructured surface, associated with a peculiar behaviour with respect to the other bases during the dehydration process, which can be effectively exploited for diagnostic purposes in DNA lesions studies.

**Figure 5:**
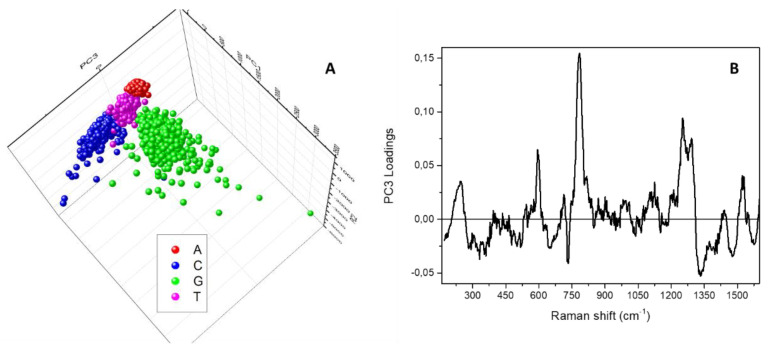
(**A**) Three-dimensional score plot resulting from the Principal Component Analysis performed on the spectra composing the Raman map acquired on adenine (red squares), cytosine (blue circles), guanine (green upward triangles) and thymine (magenta downward triangles) samples. (**B**) Spectral loadings corresponding to the third principal component, PC3.

Nevertheless, it is possible to use a different representation of the results of the PCA analysis to also separately identify and examine the clusters composed of the data coming from A, C and T sample maps. To this aim, Figure 5A reports a three-dimensional visualisation of the PCA scores obtained by using the first three principal components. The clouds appearing superposed in Figure 4A, are distributed and distinguishable along the third component PC3, whose spectral loadings are shown in Figure 5B. In this case, the relevant parameters coming from the statistical analysis are the main intense bands ascribable to the ring stretching and breathing modes of the bases, locating the A cluster in the PC3 negative half-plane (negative loading at about 735 cm^−1^) and the C cluster in the PC3 positive half-plane (positive loading at about 785 cm^−1^).

## 4. Conclusions

This study investigated the possibility of using a novel sensing approach, based on the SERS capability of a disordered array of Ag-covered silicon nanowires to analyse the characteristic Raman signal of the four DNA bases, at concentrations as low as 10 ng/µL, and study their specific way of interacting with the nanostructured substrate. For each sample, directly dropped and adsorbed onto the substrate, it was possible to find a characteristic spectrum—establishing a spectroscopic library of the four nucleotides for identification. To validate the library, a mixture containing the four bases was also studied. The spectra composing the acquired Raman maps have then been compared by Principal Component Analysis, finding a distinctive and amplified interaction of guanine, the base that is most susceptible to oxidation, with the nanostructured surface, associated with a peculiar behaviour with respect to the other bases during the dehydration process. This characteristic feature, evidenced by the proposed methodology, can explain the recently revealed diverse Raman response of cancer and normal genomic DNA deposited on the Ag/SiNWs, which is ascribed to mechanical deformation and base lesions on the backbone of the oxidised molecule. Finally, the low-cost and low-temperature fabrication technology of the Ag/SiNWs, compatible with non-conventional supports like microscope glass slides, can be easily implemented in lab-on-chips and point of care devices. Thus, the proposed platform, which also proved to be highly standardised and reproducible, will lay the foundations for a new reliable, simple, rapid and label-free DNA analysis technology, alternative to sequencing, that could be effectively used to develop therapies capable of acting at the molecular level, and diagnostic methods of high sensitivity and precision.

## Figures and Tables

**Table 1:** Peak position and possible assignment for the main features identified in the average Raman spectra of Figure 1A,B for adenine (A), guanine (G), cytosine (C) and thymine (T). Assignments follow refs 25, 26 and 27.

Band Position (cm^−1^)	Assignment
735	A, Ring breath
1329	A, C-N str, C-H bend
651	G, Ring str
959	G, 5-ring def
1322	G, ring str C-N, C-C str, C-H bend, NH2 rock
1360	G, N-H bend, C-N str
1384	G, ring str C-N, C-C str, NH2 rock, N-H bend
1460	G, ring str C-N, C-H and N-H bend
1577	G, NH2 sciss, C-N str
787	C, ring breath
1248	C, ring str C-N
1291	C, ring str C-N
1524	C, N-C str
776	T, ring breath

str = stretching, breath = breathing, bend = bending, sciss = scissoring, def = deformation.
